# The *TECTA* mutation R1890C is identified as one of the causes of genetic hearing loss: a case report

**DOI:** 10.1186/s12881-019-0775-1

**Published:** 2019-04-01

**Authors:** Gi-Sung Nam, John Hoon Rim, Jae Young Choi, Heon Yung Gee, Jong Rak Choi, Seung-Tae Lee, Jinsei Jung

**Affiliations:** 1 Department of Otorhinolaryngology, Yonsei University College of Medicine, Seoul, 03722 Republic of Korea; 2 Department of Pharmacology, Brain Korea 21 PLUS Project for Medical Sciences, Yonsei University College of Medicine, Seoul, 03722 Republic of Korea; 3 Department of Laboratory Medicine, Yonsei University College of Medicine, 50-1 Yonsei-ro Seodaemun-gu, Seoul, 03722 Republic of Korea; 4 Department of Medicine, Yonsei University Graduate School of Medicine, Seoul, 03722 Republic of Korea

**Keywords:** TECTA, Tectorin, Non-syndromic hearing loss, Congenital mild hearing loss, ACMG guideline

## Abstract

**Background:**

Many mutations in the α-tectorin gene (*TECTA*) have been reported to cause non-syndromic hearing loss (NSHL) in either a dominant or recessive inheritance pattern. Among the identified *TECTA* mutations, H1400Y has been associated with NSHL in two independent studies. However, its exact role in contributing to genetic hearing loss remains elusive.

**Case presentation:**

We herein report the whole-exome sequencing of a proband presenting with prelingual, non-progressive, mild-to-moderate hearing loss in a simplex family. By using trio-based whole-exome sequencing, we found two heterozygous mutations of R1890C and H1400Y in the ZP and ZA domains of *TECTA*, respectively. R1890C, previously reported as a pathogenic autosomal dominant mutation of genetic hearing loss, was found to be inherited in a de novo pattern, causing hearing loss in the proband. By contrast, H1400Y was not segregated in this family, and one family member with normal hearing also carried the H1400Y mutation.

**Conclusion:**

According to the hearing loss-specific American College of Medical Genetics and Genomics (ACMG) guidelines, we conclude that H1400Y should be disqualified as a cause of genetic hearing loss. True pathogenic variants causing genetic hearing loss should be more deliberately reported in accordance with ACMG guidelines.

## Background

The tectorial membrane is an extracellular matrix overlying the organ of Corti. By making contact with the stereocillia of specialized sensory hair cells, the tectorial membrane plays an important role in inducing the movement of these hair cells via intracochlear sound transmission [1]. The *TECTA* gene encodes α-tectorin, a protein of 2156 amino acid residues, that is a major component of the non-collagenous glycoproteins comprising the tectorial membrane [2]. *TECTA* is known as a causative gene of both autosomal dominant (DFNA8/A12) and autosomal recessive (DFNB21) non-syndromic sensorineural hearing loss (NSHL) [3, 4]. α-Tectorin is composed of several functional domains, including the entactin domain, four von Willebrand factor-like type D domains in the zonadhesin (ZA) domain, and the C-terminal zona pellucida (ZP) domain [5]. Autosomal dominant mutations in *TECTA* result in a broad spectrum of hearing loss depending on the domain in which the mutation occurs [5, 6], whereas the recessive mutations in *TECTA* cause mid-frequency hearing loss with a cookie-bite or U-shape audiometric configuration [7, 8]. Analysis of genotype–phenotype correlations indicate that missense mutations in the ZP domain primarily affect mid-frequency hearing loss while mutations in the ZA domain affect high-frequency hearing loss [9]. Despite several reports indicating an association of *TECTA* mutations with hereditary hearing loss, the precise molecular mechanism remains unclear. Furthermore, considering that *TECTA* mutations account for 4% of all cases of autosomal dominant NSHL (ADNSHL), *TECTA-*related ADNSHL should be regarded as one of the most frequent subtypes of ADNSHL [9].

Here, we report a 4-year-old Korean girl with non-progressive, prelingual, mid-frequency, and mild-to-moderate hearing loss, who had two missense mutations in the ZP and ZA domains of *TECTA*. Based on familial segregation analyses, we concluded that R1890C is a causative mutation for hereditary hearing loss, whereas H1400Y is not.

## Case presentation

With approval from the Institutional Review Board of the Severance Hospital, Yonsei University Health System (Seoul, Republic of Korea), a three-generation pedigree was obtained from the family by genetic counseling. The family comprised 11 individuals, four of whom participated in the study. The four members from three generations of the family were evaluated at Yonsei University Severance Hospital. For phenotype evaluation, we reviewed the medical and developmental history, and conducted physical examinations, pure tone audiometry, and a speech evaluation.

**Fig. 1:**
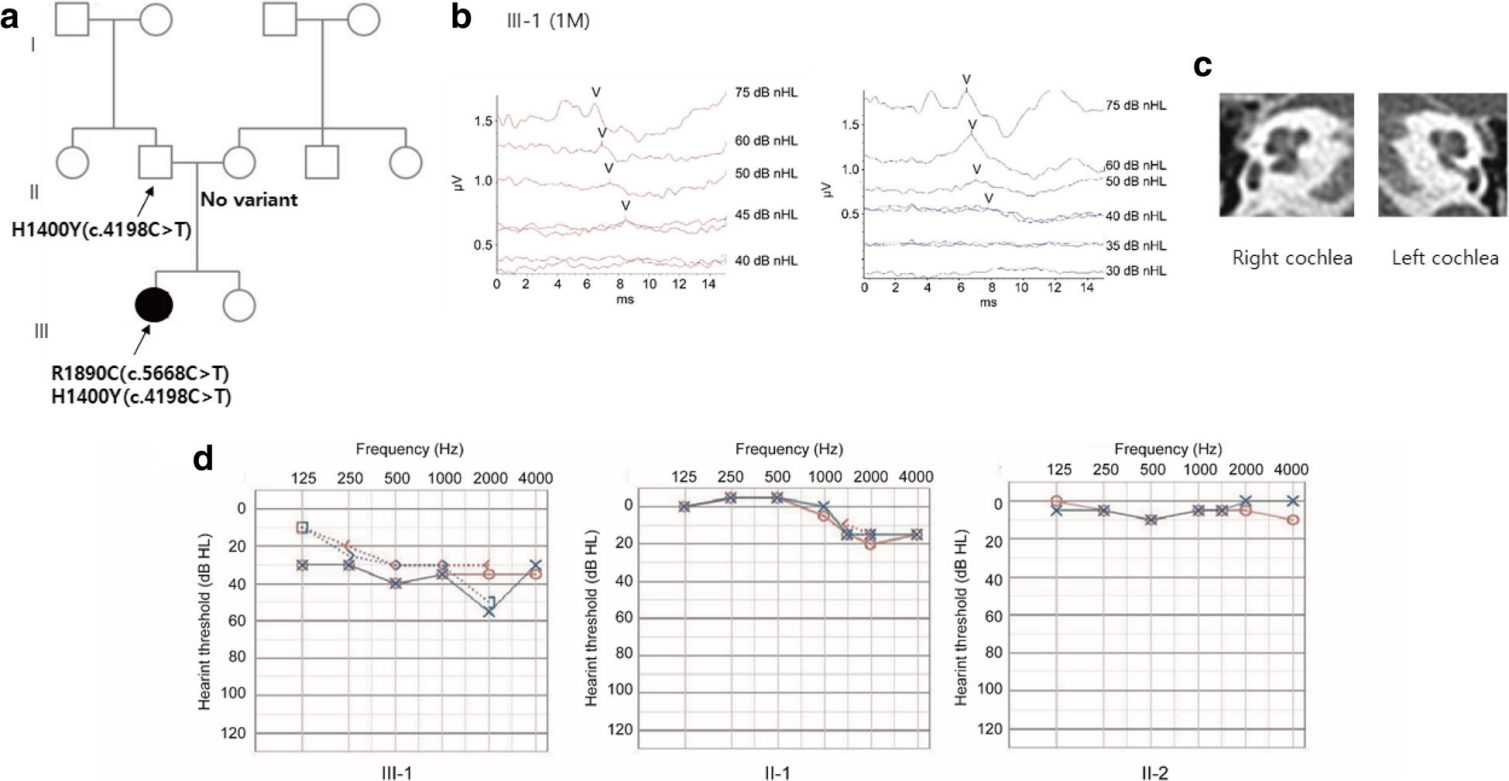
**a** Pedigree of the family with a de novo mutation causing hearing loss in the proband (filled circle). **b** Auditory brainstem response (ABR) of the proband (III-1) at 1 month after birth. **c** Temporal bone computed tomogram of the inner ear (III-1) **d** Pure-tone audiogram of the father (II-1), mother (II-2), and proband (III-1). Red and blue indicate thresholds for the right and left ears, respectively. Solid and dashed lines refer to air- and bone-conduction, respectively

The proband (III-1) was a 4-year-old female of family YUHL-165 who failed to pass the neonate hearing screening at birth and was thus referred to our department for diagnostic auditory brain stem response (ABR) testing. There was no family history related to hearing loss (Fig. 1a). She had no other risk factor of hearing loss and her ABR threshold was determined to be 45 dB nHL on the right and 40 dB nHL on the left. According to the national guideline of neonate hearing loss, we decided to perform the ABR again 3 months later and found no significant change in the ABR threshold (Fig. 1b). The temporal bone computed tomogram showed no inner ear anomaly (Fig. 1c). Audiogram performed at 4 years of follow-up revealed a mild to moderate flat shape in both ears and did not show any progression of hearing loss compared with the previously determined ABR threshold. She also did not respond in the DPOAE test, which indicates prelingual, non-progressive, mild-to-moderate sensorineural hearing loss bilaterally. All other family members had normal hearing (Fig. 1d).

**Fig. 2:**
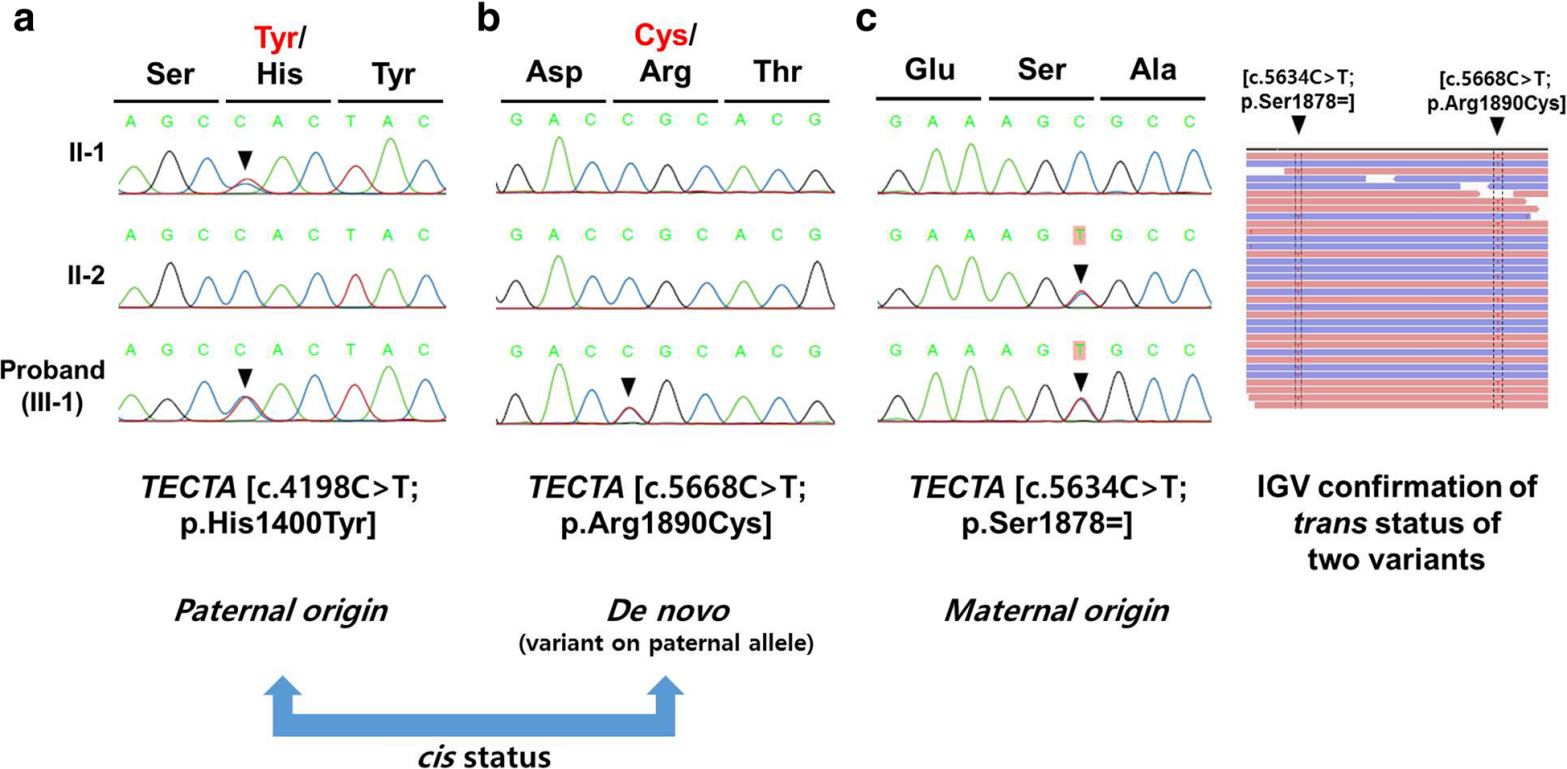
Electropherograms for the family YUHL-165 showing the heterozygous R1890C and H1400Y mutations of *TECTA* in the proband and only the H1400Y mutation in the father. **a** Segregation of the *TECTA* gene showing the H1400Y substitution. **b** Segregation of the *TECTA* gene showing the R1890C substitution. **c** Use of synonymous variant in the *TECTA* gene for cis/trans status determination of the two missense variants

To identify the genetic cause of hearing loss in this family, next-generation sequencing (NGS) was performed. Among the 2368 variants in 182 known hearing loss genes detected in our pipeline, 11 were manually reviewed for pathogenicity determination since their allele frequencies in the population database were under 0.01 (1%). Among these 11 variants in 10 hearing loss genes, two heterozygous missense variants in the *TECTA* gene were identified. We analyzed Sanger sequencing results and NGS alignment results of the R1890C variant and its adjacent synonymous variant. As the c.5634C > T synonymous variant was inherited from the mother and therefore located on the maternal allele, the cis/trans status of the c.5634C > T variant and de novo R1890C variant was revealed by NGS fragment alignment with the Integrative Genomics Viewer (IGV). Since the two variants were in trans, the de novo R1890C variant was confirmed to be located on the paternal allele. Therefore, both the H1400Y and R1890C variants were located on the paternal allele, suggesting a cis status of the two variants (Fig. 2).

The minor allele frequencies of each variant were determined in presumptively normal hearing population databases, including ESP6500, 1000 Genomes Project, and the Korean database (National Biobank of Korea, NBK) (Table 1). The variant R1890C was absent from control individuals, which translates into a status of “PM2” (i.e., absent in population databases) according to the American College of Medical Genetics and Genomics (ACMG) guideline. However, the other variant, H1400Y, is present at a relatively low allele frequency in controls and can be translated into “BS2” (i.e., observation in controls inconsistent with disease penetrance) because the hearing of the father with H1400Y was normal. From the perspective of in silico predictions, both variants were equally qualified to receive the status “PP3” (i.e., multiple computational evidences), with five out of the eight algorithms suggesting a deleterious effect (Tables 1 and 2). An extensive literature review with a search of the Human Gene Mutation Database (HGMD), Deafness Variation Database, ClinVar, and PubMed databases showed that each variant has been described in two reports; thus, both variants qualified for category “PP5” (i.e., recent reputable source without functional evidence). Considering all of these lines of evidence together, the two missense variants were suspected to additively contribute to the phenotype of hearing loss with an autosomal recessive mode of inheritance (DFNB21).

**Table 1:** Mutational profiles of *TECTA* variants detected in the proband

Gene	Nucleotide change	Amino acid change	Depth	Conservation				Minor allele frequency			In silico analysis							
				Mm	Gg	Xt	Dr	gnomAD_ all	gnomAD_ EAS	NBK	SIFT	Polyphen-2	Mutation Taster	Mutation Assessor	LRT	FATHMM	RadialSVM	CADD score
*TECTA*	c.5668C > T	p.Arg1890Cys	2571	Arg	Arg	Arg	Arg	No	No	No	D (0)	D (0.963)	D (1)	D (1.385)	D (0)	D (−1.62)	D (0.383)	19.99
	c.4198C > T	p.His1400Tyr	382	His	His	His	Gln	0.0002166	0.003682	0.00503778	D (0)	D (0.793)	D (0.823)	N (0.62)	D (0)	D (−2.69)	D (0.372)	22.7

Abbreviations: *Arg* (R) arginine, *CADD* combined annotation-dependent depletion, *Cys* (C) cysteine, *D* deleterious or damaging prediction results, *Dr*. *Danio rerio*, *EAS* east Asian, *Gg Gallus gallus*, *Gln* (Q) glutamine, *His* (H) histidine, *LRT* likelihood ratio test, *Mm Mus musculus*, *N* neutral or non-deleterious prediction results, *NBK* national biobank of Korea, *Polyphen-2* polymorphism phenotyping v2, *RadialSVM* radial support vector machine, *SIFT* sorting tolerant from intolerant, *Tyr* (Y) tyrosine, *Xt* Xenopus tropicalis

**Table 2:** Pathogenicity evaluation of two variants based on in-depth ACMG guideline application

Gene	Variant	Literature reported the variant				In-depth ACMG guideline interpretation					
		Literature 1		Literature 2		Original ACMG guideline application			Hearing loss-specified ACMG guideline application^a^		
		PMID (year)	Evidential level	PMID (year)	Evidential level	Pathogenic components	Benign components	Final Result	Pathogenic components	Benign components	Final Result
*TECTA*	R1890C	16718611 (2006)	Clinical report of 1 family with segregation	28946916 (2017)	Clinical report of 3 families with segregation	PS2, PM2, PP3, PP5	no component	Likely pathogenic	PS2_Moderate, PS4_Supporting, PM2, PP1_Strong, PP3	no component	Pathogenic
	H1400Y	22718023 (2012)	Clinical report of 1 family with segregation	23967202 (2013)	Simple clinical report without segregation	PP3, PP5	BS2	Variant of uncertain significance	PP3	BS1, BS2	Benign

^a^Specified ACMG hearing loss rules suggested by the ClinGen Hearing Loss Clinical Domain Working Group were applied [15]

Abbreviations: *ACMG* American College of Medical Genetics and Genomics, *PMID* pubmed ID, *PS* pathogenic strong, *PM* pathogenic moderate, *PP* pathogenic support

PS2, 1 proven de novo occurrence; PM2, absent in population databases; PP3, multiple computational evidence; PP5, recent reputable source without functional evidence; BS2, observation on in controls inconsistent with disease penetrance; PS2_Moderate, 1 proven de novo occurrence (phenotype consistent but not specific to gene); PS4_Supporting, Autosomal dominant: ≥2 probands with variant, and variant meets; PM2, Absent/rare in population databases; PP1_Strong, Segregation in one affected relative for recessive and two affected relatives for dominant; BS1, MAF of ≥0.003 (0.3%) for autosomal recessive; MAF of ≥0.0002 (0.02%) for autosomal dominant. Likely benign, provided there is no conflicting evidence

Among the two candidate variants in the *TECTA* gene, H1400Y was revealed to be inherited from the proband’s father in the trio study (Fig. 2). However, R1890C was not observed in either parent, indicating de novo status. Although this result allowed us to assign the status “PS2” (i.e., de novo, paternity and maternity confirmed) to the R1890C variant (Table 2), it also indicated the requirement of further phenotypic analysis of the parents since two scenarios were possible: 1) R1890C and H1400Y could act as two additive variants in an autosomal recessive inheritance pattern, or 2) R1890C could independently act as a single mutation in an autosomal dominant inheritance pattern. According to previous reports, dominant *TECTA* mutations are generally associated with stable and post-lingual progressive hearing loss, whereas recessive *TECTA* mutations are generally associated with pre-lingual stable hearing loss. Therefore, phenotype-based genotype interpretation was further applied for identification of true variants.

According to the original ACMG guideline interpretation, the variants R1890C and H1400Y could be classified as “likely pathogenic” and “variant of uncertain significance”, respectively (Table 2). These results could not determine whether the *TECTA* mutation is inherited in an autosomal dominant or recessive mode. However, application of the updated hearing loss-specified ACMG guideline maximized the evidential weight for each component, thereby allowing us to designate R1890C as “pathogenic” rather than “likely pathogenic”, and H1400Y as “benign” rather than “variant of uncertain significance” (Table 2).

## Discussion and conclusion

ADNSHL has both genetically and clinically heterogeneous characteristics, and 24 associated genes have been identified to date [10]. *TECTA* is one of the most common genes responsible for ADNSHL and has been associated with various types of hearing loss in terms of progression and affected frequency range [3, 4]. The position of the mutations in either the ZP or ZA domain of the encoded α-tectorin protein, and the spectrum of the amino acid substitutions determine the pathogenic characteristics of the *TECTA* mutation. Currently established genotype-phenotype correlations in *TECTA* indicate that mutations in the ZP and ZA domains are generally related to mid- and high-frequency hearing loss, respectively [4–6, 11]. However, in 2011, Hildebrand et al. [9] challenged these genotype-phenotype correlations based on the results of a large cohort study indicating that ZA domain mutations are also associated with mid-frequency hearing loss. It is possible that if mutations of both domains similarly affect processing of α-tectorin, the ZA mutation might disrupt polypeptide assembly, thereby having a distinct impact on frequency ranges [12].

Two *TECTA* missense mutations, R1890C and H1400Y, were detected in the proband of the present trio study. The variant H1400Y is located in exon 12 corresponding to the ZA domain of α-tectorin, whereas R1890C is located in the ZP domain. R1890C was previously reported as a pathogenic mutation with a high level of evidence [11]. However, H1400Y was not clearly proven to be pathogenic in two previous studies [13, 14]. One study involved a Japanese family with heterozygous H1400Y and T1866 M mutations of *TECTA*. Four of the five affected family members were evaluated, all of whom demonstrated bilateral, mild to moderate symmetric sensorineural hearing loss, affecting the mid frequencies. Interestingly, the H1400Y mutation was present in the cis-form with the other deleterious mutation T1866M. The authors suggested that T1866M was likely the causative mutation of the hearing impairment in this family, although the effect of H1400Y could not be completely ruled out at that time [14]. In another report, H1400Y was identified in a patient with congenital severe hearing loss, but no familial segregation study was performed [13]. In 2006, Plantinga et al. [11] first reported the R1890C mutation, which was combined with the T83M mutation in α-tectorin, in a Dutch family with autosomal dominant mid-frequency hearing loss. R1890C is located in the ZP domain of α-tectorin, whereas T83M could not be aligned to a specific domain. Considering the phenotype, position of the mutation, and molecular mechanism of cysteine mutation, the authors suggested that the impact of the T83M mutation is negligible, whereas R1890C is likely to be causative for genetic hearing loss. Based on the incongruent genotype-phenotype results of these two studies, we assumed that H1400Y is unlikely to be pathogenic. Supporting this assumption, the proband of the present study had two missense mutations and the audiogram showed mild-to-moderate hearing loss, while her father with only the H1400Y mutation showed a completely normal audiogram. Furthermore, the other variant R1890C was revealed to be a de novo mutation based on the familial segregation study, suggesting a high likelihood of pathogenicity.

Interestingly, all of the mutations of *TECTA* associated with ADNSHL identified to date are missense variants, while nonsense mutations are associated with an autosomal recessive pattern of inheritance. As mentioned above, the specific pattern of hearing loss caused by a *TECTA* mutation depends on the affected domains. Missense mutations in the ZP and ZA domains are generally related to mid- and high-frequency hearing loss, respectively. However, the proband in this study showed a flat-type audiogram, which seems to affect the high frequency. There are several possibilities to explain this finding. First, the reliability of the proband’s pure tone audiometry could be relatively poor due to her young age. Second, the synergistic effect of the two mutations cannot be completely excluded. Third, the post-lingual hearing loss may be due to a genetic cause inherited from her father. Therefore, accurate follow-up hearing testing will be needed for this family.

Unfortunately, there has been no in vitro functional assay to evaluate the mechanism by which *TECTA* mutations affect hearing loss. In this case, other available lines of evidence such as those derived from segregation studies should be incorporated to pinpoint true genetic causes. The combination of genetic and segregation analyses can help to provide several counseling strategies for determining the progression, prediction, and therapeutic application for this young patient.

Recent improvements of variant interpretation guidelines, including the hearing loss-specified ACMG guideline established by the ClinGen Working Group, advocated our findings. Compared to the original ACMG guideline, which provided inconclusive results even when conducting a familial segregation study, the optimized ACMG guideline maximized the pathogenicity evaluation results and pinpointed the true pathogenic mutation. As *TECTA* is one of the major contributing causative genes of hearing loss, accurate application of hearing loss-specified ACMG rules is expected to enhance classification of all of the variants identified to date.

In conclusion, we identified the R1890C mutation in the *TECTA* gene as a genetic cause of hearing loss, whereas the H1400Y variant was determined to be an innocent bystander. To the best of our knowledge, this is the first study to provide clear evidence as to whether or not H1400Y is responsible for hearing loss, including results from a familial segregation study and hearing loss-specified ACMG guideline application. Therefore, this study is expected to contribute to gaining a better understanding of the *TECTA* gene mutation spectrum.

## Abbreviations

ACMG: American College of Medical Genetics and Genomics
NGS: next-generation sequencing
NSHL: non-syndromic hearing loss
